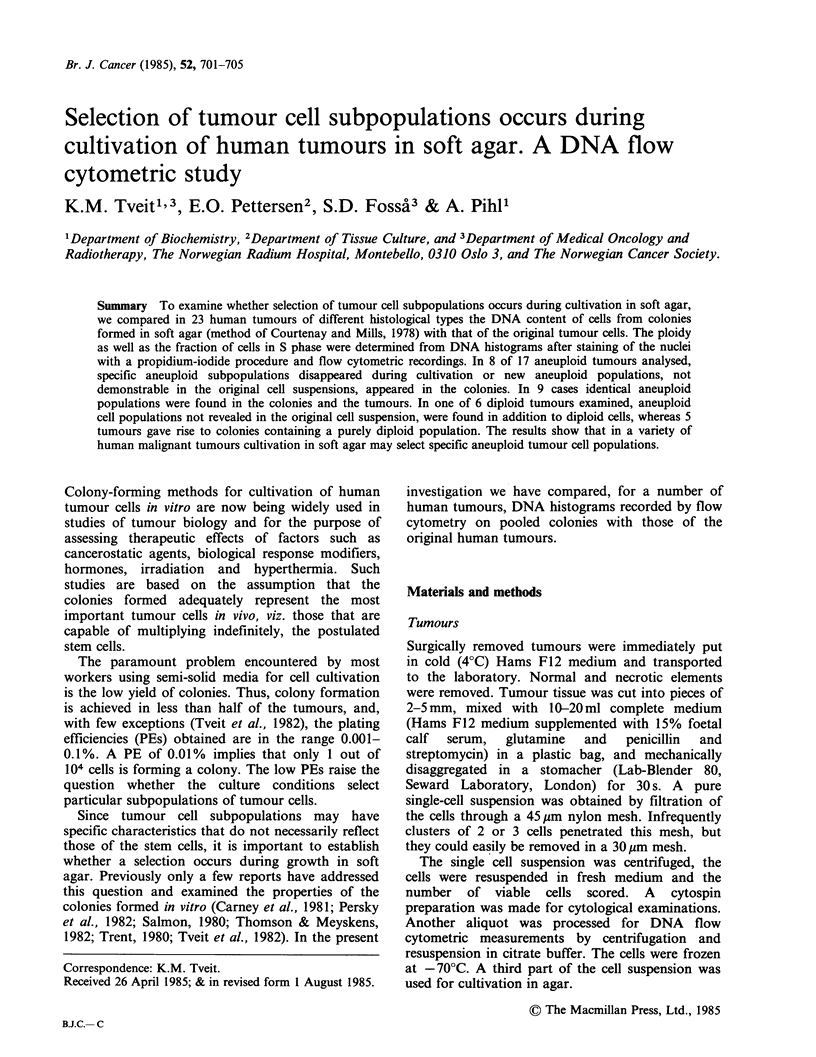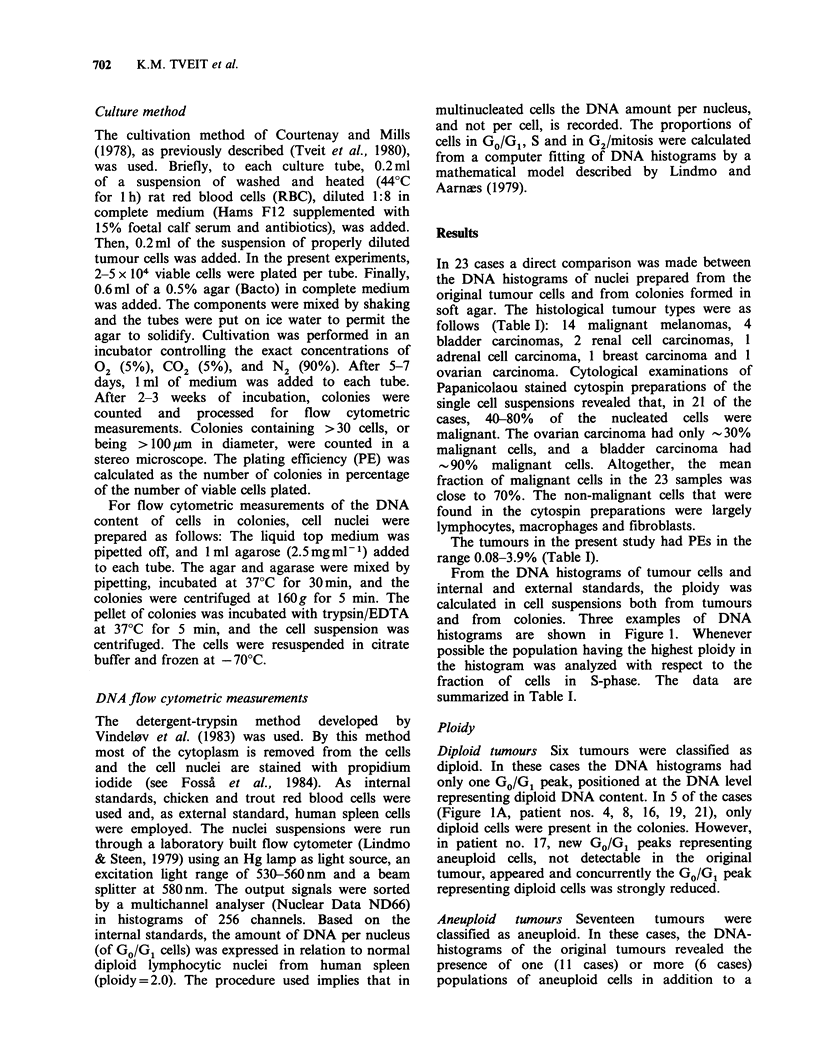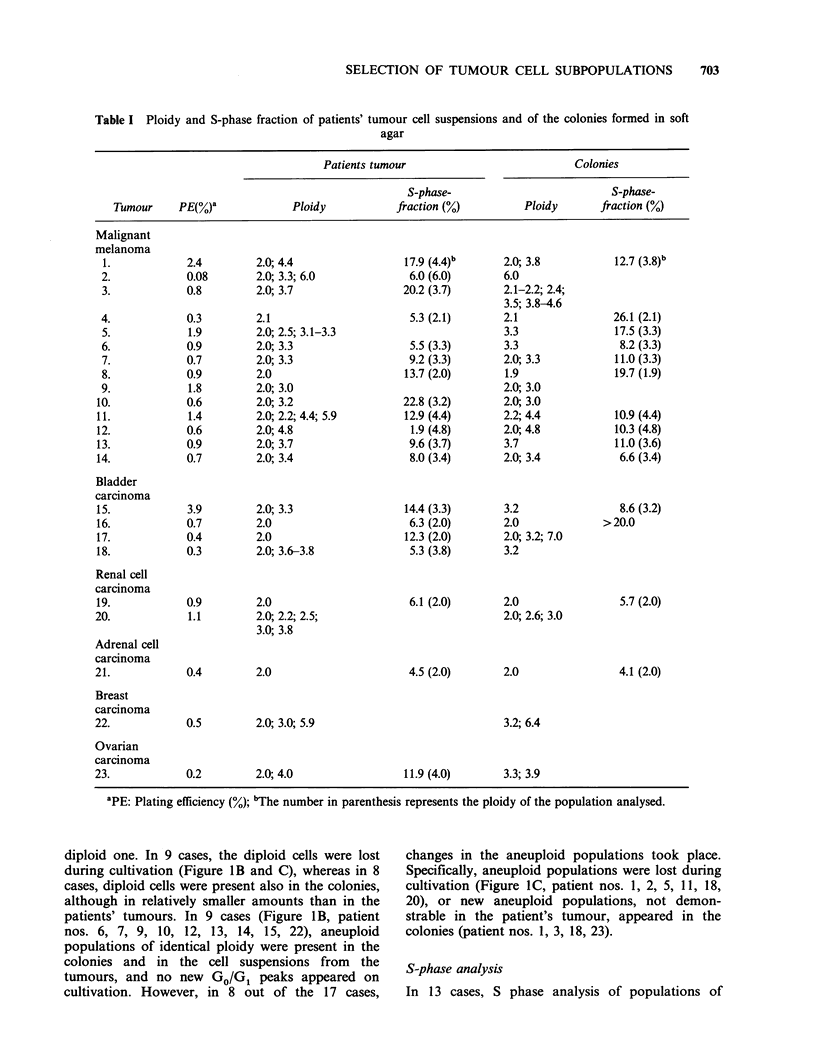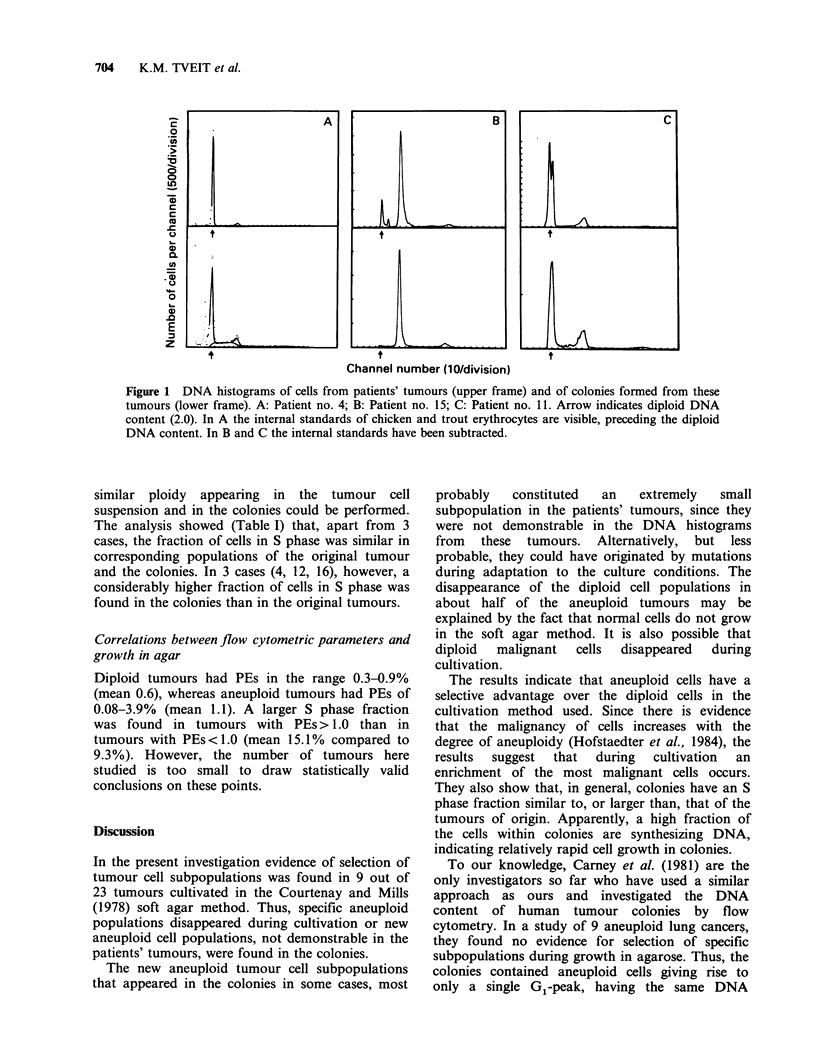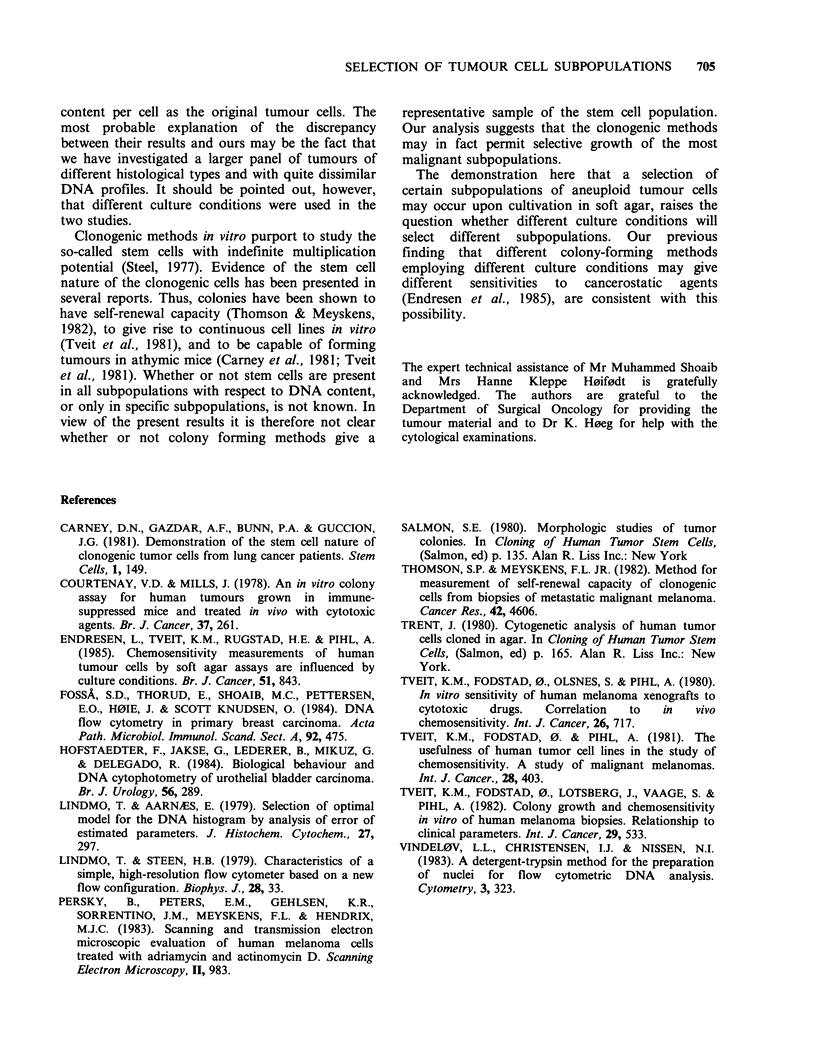# Selection of tumour cell subpopulations occurs during cultivation of human tumours in soft agar. A DNA flow cytometric study.

**DOI:** 10.1038/bjc.1985.246

**Published:** 1985-11

**Authors:** K. M. Tveit, E. O. Pettersen, S. D. Fosså, A. Pihl

## Abstract

To examine whether selection of tumour cell subpopulations occurs during cultivation in soft agar, we compared in 23 human tumours of different histological types the DNA content of cells from colonies formed in soft agar (method of Courtenay and Mills, 1978) with that of the original tumour cells. The ploidy as well as the fraction of cells in S phase were determined from DNA histograms after staining of the nuclei with a propidium-iodide procedure and flow cytometric recordings. In 8 of 17 aneuploid tumours analysed, specific aneuploid subpopulations disappeared during cultivation or new aneuploid populations, not demonstrable in the original cell suspensions, appeared in the colonies. In 9 cases identical aneuploid populations were found in the colonies and the tumours. In one of 6 diploid tumours examined, aneuploid cell populations not revealed in the original cell suspension, were found in addition to diploid cells, whereas 5 tumours gave rise to colonies containing a purely diploid population. The results show that in a variety of human malignant tumours cultivation in soft agar may select specific aneuploid tumour cell populations.


*Br. J. Cancer* (1985), **52**, 701–705

### Selection of tumour cell subpopulations occurs during cultivation of human tumours in soft agar. A DNA flow cytometric study

K.M. Tveit[1,3], E.O. Pettersen[2], S.D. Fosså[3] & A. Pihl[1]

[1]*Department of Biochemistry,* [2]*Department of Tissue Culture, and* [3]*Department of Medical Oncology and Radiotherapy, The Norwegian Radium Hospital, Montebello, 0310 Oslo 3, and The Norwegian Cancer Society.*

**Summary** To examine whether selection of tumour cell subpopulations occurs during cultivation in soft agar, we compared in 23 human tumours of different histological types the DNA content of cells from colonies formed in soft agar (method of Courtenay and Mills, 1978) with that of the original tumour cells. The ploidy as well as the fraction of cells in S phase were determined from DNA histograms after staining of the nuclei with a propidium-iodide procedure and flow cytometric recordings. In 8 of 17 aneuploid tumours analysed, specific aneuploid subpopulations disappeared during cultivation or new aneuploid populations, not demonstrable in the original cell suspensions, appeared in the colonies. In 9 cases identical aneuploid populations were found in the colonies and the tumours. In one of 6 diploid tumours examined, aneuploid cell populations not revealed in the original cell suspension, were found in addition to diploid cells, whereas 5 tumours gave rise to colonies containing a purely diploid population. The results show that in a variety of human malignant tumours cultivation in soft agar may select specific aneuploid tumour cell populations.

Colony-forming methods for cultivation of human tumour cells *in vitro* are now being widely used in studies of tumour biology and for the purpose of assessing therapeutic effects of factors such as cancerostatic agents, biological response modifiers, hormones, irradiation and hyperthermia. Such studies are based on the assumption that the colonies formed adequately represent the most important tumour cells *in vivo, viz.* those that are capable of multiplying indefinitely, the postulated stem cells.

The paramount problem encountered by most workers using semi-solid media for cell cultivation is the low yield of colonies. Thus, colony formation is achieved in less than half of the tumours, and, with few exceptions (Tveit *et al.*, 1982), the plating efficiencies (PEs) obtained are in the range 0.001–0.1%. A PE of 0.01% implies that only 1 out of $10^4$ cells is forming a colony. The low PEs raise the question whether the culture conditions select particular subpopulations of tumour cells.

Since tumour cell subpopulations may have specific characteristics that do not necessarily reflect those of the stem cells, it is important to establish whether a selection occurs during growth in soft agar. Previously only a few reports have addressed this question and examined the properties of the colonies formed *in vitro* (Carney *et al.*, 1981; Persky *et al.*, 1982; Salmon, 1980; Thomson & Meyskens, 1982; Trent, 1980; Tveit *et al.*, 1982). In the present investigation we have compared, for a number of human tumours, DNA histograms recorded by flow cytometry on pooled colonies with those of the original human tumours.

Correspondence: K.M. Tveit.
Received 26 April 1985; & in revised form 1 August 1985.

### Materials and methods

### *Tumours*

Surgically removed tumours were immediately put in cold (4°C) Hams F12 medium and transported to the laboratory. Normal and necrotic elements were removed. Tumour tissue was cut into pieces of 2–5 mm, mixed with 10–20 ml complete medium (Hams F12 medium supplemented with 15% foetal calf serum, glutamine and penicillin and streptomycin) in a plastic bag, and mechanically disaggregated in a stomacher (Lab-Blender 80, Seward Laboratory, London) for 30 s. A pure single-cell suspension was obtained by filtration of the cells through a 45 $\mu$m nylon mesh. Infrequently clusters of 2 or 3 cells penetrated this mesh, but they could easily be removed in a 30 $\mu$m mesh.

The single cell suspension was centrifuged, the cells were resuspended in fresh medium and the number of viable cells scored. A cytospin preparation was made for cytological examinations. Another aliquot was processed for DNA flow cytometric measurements by centrifugation and resuspension in citrate buffer. The cells were frozen at −70°C. A third part of the cell suspension was used for cultivation in agar.

© The Macmillan Press, Ltd., 1985

*Culture method*

The cultivation method of Courtenay and Mills (1978), as previously described (Tveit *et al.*, 1980), was used. Briefly, to each culture tube, 0.2 ml of a suspension of washed and heated (44°C for 1 h) rat red blood cells (RBC), diluted 1:8 in complete medium (Hams F12 supplemented with 15% foetal calf serum and antibiotics), was added. Then, 0.2 ml of the suspension of properly diluted tumour cells was added. In the present experiments, $2–5 \times 10^4$ viable cells were plated per tube. Finally, 0.6 ml of a 0.5% agar (Bacto) in complete medium was added. The components were mixed by shaking and the tubes were put on ice water to permit the agar to solidify. Cultivation was performed in an incubator controlling the exact concentrations of $O_2$ (5%), $CO_2$ (5%), and $N_2$ (90%). After 5–7 days, 1 ml of medium was added to each tube. After 2–3 weeks of incubation, colonies were counted and processed for flow cytometric measurements. Colonies containing >30 cells, or being >100 $\mu$m in diameter, were counted in a stereo microscope. The plating efficiency (PE) was calculated as the number of colonies in percentage of the number of viable cells plated.

For flow cytometric measurements of the DNA content of cells in colonies, cell nuclei were prepared as follows: The liquid top medium was pipetted off, and 1 ml agarose (2.5 mg ml$^{-1}$) added to each tube. The agar and agarase were mixed by pipetting, incubated at 37°C for 30 min, and the colonies were centrifuged at 160 $g$ for 5 min. The pellet of colonies was incubated with trypsin/EDTA at 37°C for 5 min, and the cell suspension was centrifuged. The cells were resuspended in citrate buffer and frozen at −70°C.

*DNA flow cytometric measurements*

The detergent-trypsin method developed by Vindeløv *et al.* (1983) was used. By this method most of the cytoplasm is removed from the cells and the cell nuclei are stained with propidium iodide (see Fosså *et al.*, 1984). As internal standards, chicken and trout red blood cells were used and, as external standard, human spleen cells were employed. The nuclei suspensions were run through a laboratory built flow cytometer (Lindmo & Steen, 1979) using an Hg lamp as light source, an excitation light range of 530–560 nm and a beam splitter at 580 nm. The output signals were sorted by a multichannel analyser (Nuclear Data ND66) in histograms of 256 channels. Based on the internal standards, the amount of DNA per nucleus (of $G_0/G_1$ cells) was expressed in relation to normal diploid lymphocytic nuclei from human spleen (ploidy = 2.0). The procedure used implies that in multinucleated cells the DNA amount per nucleus, and not per cell, is recorded. The proportions of cells in $G_0/G_1$, S and in $G_2$/mitosis were calculated from a computer fitting of DNA histograms by a mathematical model described by Lindmo and Aarnæs (1979).

### Results

In 23 cases a direct comparison was made between the DNA histograms of nuclei prepared from the original tumour cells and from colonies formed in soft agar. The histological tumour types were as follows (Table I): 14 malignant melanomas, 4 bladder carcinomas, 2 renal cell carcinomas, 1 adrenal cell carcinoma, 1 breast carcinoma and 1 ovarian carcinoma. Cytological examinations of Papanicolaou stained cytospin preparations of the single cell suspensions revealed that, in 21 of the cases, 40–80% of the nucleated cells were malignant. The ovarian carcinoma had only ~30% malignant cells, and a bladder carcinoma had ~90% malignant cells. Altogether, the mean fraction of malignant cells in the 23 samples was close to 70%. The non-malignant cells that were found in the cytospin preparations were largely lymphocytes, macrophages and fibroblasts.

The tumours in the present study had PEs in the range 0.08–3.9% (Table I).

From the DNA histograms of tumour cells and internal and external standards, the ploidy was calculated in cell suspensions both from tumours and from colonies. Three examples of DNA histograms are shown in Figure 1. Whenever possible the population having the highest ploidy in the histogram was analyzed with respect to the fraction of cells in S-phase. The data are summarized in Table I.

*Ploidy*

*Diploid tumours* Six tumours were classified as diploid. In these cases the DNA histograms had only one $G_0/G_1$ peak, positioned at the DNA level representing diploid DNA content. In 5 of the cases (Figure 1A, patient nos. 4, 8, 16, 19, 21), only diploid cells were present in the colonies. However, in patient no. 17, new $G_0/G_1$ peaks representing aneuploid cells, not detectable in the original tumour, appeared and concurrently the $G_0/G_1$ peak representing diploid cells was strongly reduced.

*Aneuploid tumours* Seventeen tumours were classified as aneuploid. In these cases, the DNA-histograms of the original tumours revealed the presence of one (11 cases) or more (6 cases) populations of aneuploid cells in addition to a

**Table I** Ploidy and S-phase fraction of patients' tumour cell suspensions and of the colonies formed in soft agar

| | | *Patients tumour* | | *Colonies* | |
|---|---|---|---|---|---|
| *Tumour* | *PE(%)*[a] | *Ploidy* | *S-phase-fraction (%)* | *Ploidy* | *S-phase-fraction (%)* |
| Malignant melanoma | | | | | |
| 1. | 2.4 | 2.0; 4.4 | 17.9 (4.4)[b] | 2.0; 3.8 | 12.7 (3.8)[b] |
| 2. | 0.08 | 2.0; 3.3; 6.0 | 6.0 (6.0) | 6.0 | |
| 3. | 0.8 | 2.0; 3.7 | 20.2 (3.7) | 2.1–2.2; 2.4; 3.5; 3.8–4.6 | |
| 4. | 0.3 | 2.1 | 5.3 (2.1) | 2.1 | 26.1 (2.1) |
| 5. | 1.9 | 2.0; 2.5; 3.1–3.3 | | 3.3 | 17.5 (3.3) |
| 6. | 0.9 | 2.0; 3.3 | 5.5 (3.3) | 3.3 | 8.2 (3.3) |
| 7. | 0.7 | 2.0; 3.3 | 9.2 (3.3) | 2.0; 3.3 | 11.0 (3.3) |
| 8. | 0.9 | 2.0 | 13.7 (2.0) | 1.9 | 19.7 (1.9) |
| 9. | 1.8 | 2.0; 3.0 | | 2.0; 3.0 | |
| 10. | 0.6 | 2.0; 3.2 | 22.8 (3.2) | 2.0; 3.0 | |
| 11. | 1.4 | 2.0; 2.2; 4.4; 5.9 | 12.9 (4.4) | 2.2; 4.4 | 10.9 (4.4) |
| 12. | 0.6 | 2.0; 4.8 | 1.9 (4.8) | 2.0; 4.8 | 10.3 (4.8) |
| 13. | 0.9 | 2.0; 3.7 | 9.6 (3.7) | 3.7 | 11.0 (3.6) |
| 14. | 0.7 | 2.0; 3.4 | 8.0 (3.4) | 2.0; 3.4 | 6.6 (3.4) |
| Bladder carcinoma | | | | | |
| 15. | 3.9 | 2.0; 3.3 | 14.4 (3.3) | 3.2 | 8.6 (3.2) |
| 16. | 0.7 | 2.0 | 6.3 (2.0) | 2.0 | >20.0 |
| 17. | 0.4 | 2.0 | 12.3 (2.0) | 2.0; 3.2; 7.0 | |
| 18. | 0.3 | 2.0; 3.6–3.8 | 5.3 (3.8) | 3.2 | |
| Renal cell carcinoma | | | | | |
| 19. | 0.9 | 2.0 | 6.1 (2.0) | 2.0 | 5.7 (2.0) |
| 20. | 1.1 | 2.0; 2.2; 2.5; 3.0; 3.8 | | 2.0; 2.6; 3.0 | |
| Adrenal cell carcinoma | | | | | |
| 21. | 0.4 | 2.0 | 4.5 (2.0) | 2.0 | 4.1 (2.0) |
| Breast carcinoma | | | | | |
| 22. | 0.5 | 2.0; 3.0; 5.9 | | 3.2; 6.4 | |
| Ovarian carcinoma | | | | | |
| 23. | 0.2 | 2.0; 4.0 | 11.9 (4.0) | 3.3; 3.9 | |

[a]PE: Plating efficiency (%); [b]The number in parenthesis represents the ploidy of the population analysed.

diploid one. In 9 cases, the diploid cells were lost during cultivation (Figure 1B and C), whereas in 8 cases, diploid cells were present also in the colonies, although in relatively smaller amounts than in the patients' tumours. In 9 cases (Figure 1B, patient nos. 6, 7, 9, 10, 12, 13, 14, 15, 22), aneuploid populations of identical ploidy were present in the colonies and in the cell suspensions from the tumours, and no new $G_0/G_1$ peaks appeared on cultivation. However, in 8 out of the 17 cases, changes in the aneuploid populations took place. Specifically, aneuploid populations were lost during cultivation (Figure 1C, patient nos. 1, 2, 5, 11, 18, 20), or new aneuploid populations, not demonstrable in the patient's tumour, appeared in the colonies (patient nos. 1, 3, 18, 23).

*S-phase analysis*

In 13 cases, S phase analysis of populations of

**Figure 1** DNA histograms of cells from patients' tumours (upper frame) and of colonies formed from these tumours (lower frame). A: Patient no. 4; B: Patient no. 15; C: Patient no. 11. Arrow indicates diploid DNA content (2.0). In A the internal standards of chicken and trout erythrocytes are visible, preceding the diploid DNA content. In B and C the internal standards have been subtracted.

similar ploidy appearing in the tumour cell suspension and in the colonies could be performed. The analysis showed (Table I) that, apart from 3 cases, the fraction of cells in S phase was similar in corresponding populations of the original tumour and the colonies. In 3 cases (4, 12, 16), however, a considerably higher fraction of cells in S phase was found in the colonies than in the original tumours.

*Correlations between flow cytometric parameters and growth in agar*

Diploid tumours had PEs in the range 0.3–0.9% (mean 0.6), whereas aneuploid tumours had PEs of 0.08–3.9% (mean 1.1). A larger S phase fraction was found in tumours with PEs > 1.0 than in tumours with PEs < 1.0 (mean 15.1% compared to 9.3%). However, the number of tumours here studied is too small to draw statistically valid conclusions on these points.

### Discussion

In the present investigation evidence of selection of tumour cell subpopulations was found in 9 out of 23 tumours cultivated in the Courtenay and Mills (1978) soft agar method. Thus, specific aneuploid populations disappeared during cultivation or new aneuploid cell populations, not demonstrable in the patients' tumours, were found in the colonies.

The new aneuploid tumour cell subpopulations that appeared in the colonies in some cases, most probably constituted an extremely small subpopulation in the patients' tumours, since they were not demonstrable in the DNA histograms from these tumours. Alternatively, but less probable, they could have originated by mutations during adaptation to the culture conditions. The disappearance of the diploid cell populations in about half of the aneuploid tumours may be explained by the fact that normal cells do not grow in the soft agar method. It is also possible that diploid malignant cells disappeared during cultivation.

The results indicate that aneuploid cells have a selective advantage over the diploid cells in the cultivation method used. Since there is evidence that the malignancy of cells increases with the degree of aneuploidy (Hofstaedter *et al.*, 1984), the results suggest that during cultivation an enrichment of the most malignant cells occurs. They also show that, in general, colonies have an S phase fraction similar to, or larger than, that of the tumours of origin. Apparently, a high fraction of the cells within colonies are synthesizing DNA, indicating relatively rapid cell growth in colonies.

To our knowledge, Carney *et al.* (1981) are the only investigators so far who have used a similar approach as ours and investigated the DNA content of human tumour colonies by flow cytometry. In a study of 9 aneuploid lung cancers, they found no evidence for selection of specific subpopulations during growth in agarose. Thus, the colonies contained aneuploid cells giving rise to only a single $G_1$-peak, having the same DNA

content per cell as the original tumour cells. The most probable explanation of the discrepancy between their results and ours may be the fact that we have investigated a larger panel of tumours of different histological types and with quite dissimilar DNA profiles. It should be pointed out, however, that different culture conditions were used in the two studies.

Clonogenic methods *in vitro* purport to study the so-called stem cells with indefinite multiplication potential (Steel, 1977). Evidence of the stem cell nature of the clonogenic cells has been presented in several reports. Thus, colonies have been shown to have self-renewal capacity (Thomson & Meyskens, 1982), to give rise to continuous cell lines *in vitro* (Tveit *et al.*, 1981), and to be capable of forming tumours in athymic mice (Carney *et al.*, 1981; Tveit *et al.*, 1981). Whether or not stem cells are present in all subpopulations with respect to DNA content, or only in specific subpopulations, is not known. In view of the present results it is therefore not clear whether or not colony forming methods give a representative sample of the stem cell population. Our analysis suggests that the clonogenic methods may in fact permit selective growth of the most malignant subpopulations.

The demonstration here that a selection of certain subpopulations of aneuploid tumour cells may occur upon cultivation in soft agar, raises the question whether different culture conditions will select different subpopulations. Our previous finding that different colony-forming methods employing different culture conditions may give different sensitivities to cancerostatic agents (Endresen *et al.*, 1985), are consistent with this possibility.

The expert technical assistance of Mr Muhammed Shoaib and Mrs Hanne Kleppe Høifødt is gratefully acknowledged. The authors are grateful to the Department of Surgical Oncology for providing the tumour material and to Dr K. Høeg for help with the cytological examinations.

**References**

CARNEY, D.N., GAZDAR, A.F., BUNN, P.A. & GUCCION, J.G. (1981). Demonstration of the stem cell nature of clonogenic tumor cells from lung cancer patients. *Stem Cells*, **1**, 149.

COURTENAY, V.D. & MILLS, J. (1978). An *in vitro* colony assay for human tumours grown in immune-suppressed mice and treated *in vivo* with cytotoxic agents. *Br. J. Cancer*, **37**, 261.

ENDRESEN, L., TVEIT, K.M., RUGSTAD, H.E. & PIHL, A. (1985). Chemosensitivity measurements of human tumour cells by soft agar assays are influenced by culture conditions. *Br. J. Cancer*, **51**, 843.

FOSSÅ, S.D., THORUD, E., SHOAIB, M.C., PETTERSEN, E.O., HØIE, J. & SCOTT KNUDSEN, O. (1984). DNA flow cytometry in primary breast carcinoma. *Acta Path. Microbiol. Immunol. Scand. Sect. A*, **92**, 475.

HOFSTAEDTER, F., JAKSE, G., LEDERER, B., MIKUZ, G. & DELEGADO, R. (1984). Biological behaviour and DNA cytophotometry of urothelial bladder carcinoma. *Br. J. Urology*, **56**, 289.

LINDMO, T. & AARNÆS, E. (1979). Selection of optimal model for the DNA histogram by analysis of error of estimated parameters. *J. Histochem. Cytochem.*, **27**, 297.

LINDMO, T. & STEEN, H.B. (1979). Characteristics of a simple, high-resolution flow cytometer based on a new flow configuration. *Biophys. J.*, **28**, 33.

PERSKY, B., PETERS, E.M., GEHLSEN, K.R., SORRENTINO, J.M., MEYSKENS, F.L. & HENDRIX, M.J.C. (1983). Scanning and transmission electron microscopic evaluation of human melanoma cells treated with adriamycin and actinomycin D. *Scanning Electron Microscopy*, **II**, 983.

SALMON, S.E. (1980). Morphologic studies of tumor colonies. In *Cloning of Human Tumor Stem Cells*, (Salmon, ed) p. 135. Alan R. Liss Inc.: New York

THOMSON, S.P. & MEYSKENS, F.L. JR. (1982). Method for measurement of self-renewal capacity of clonogenic cells from biopsies of metastatic malignant melanoma. *Cancer Res.*, **42**, 4606.

TRENT, J. (1980). Cytogenetic analysis of human tumor cells cloned in agar. In *Cloning of Human Tumor Stem Cells*, (Salmon, ed) p. 165. Alan R. Liss Inc.: New York.

TVEIT, K.M., FODSTAD, Ø., OLSNES, S. & PIHL, A. (1980). *In vitro* sensitivity of human melanoma xenografts to cytotoxic drugs. Correlation to *in vivo* chemosensitivity. *Int. J. Cancer*, **26**, 717.

TVEIT, K.M., FODSTAD, Ø. & PIHL, A. (1981). The usefulness of human tumor cell lines in the study of chemosensitivity. A study of malignant melanomas. *Int. J. Cancer.*, **28**, 403.

TVEIT, K.M., FODSTAD, Ø., LOTSBERG, J., VAAGE, S. & PIHL, A. (1982). Colony growth and chemosensitivity *in vitro* of human melanoma biopsies. Relationship to clinical parameters. *Int. J. Cancer*, **29**, 533.

VINDELØV, L.L., CHRISTENSEN, I.J. & NISSEN, N.I. (1983). A detergent-trypsin method for the preparation of nuclei for flow cytometric DNA analysis. *Cytometry*, **3**, 323.